# Evaluation of In-Hospital Management for Febrile Illness in Northern Tanzania before and after 2010 World Health Organization Guidelines for the Treatment of Malaria

**DOI:** 10.1371/journal.pone.0089814

**Published:** 2014-02-24

**Authors:** Andrew M. Moon, Holly M. Biggs, Matthew P. Rubach, John A. Crump, Venace P. Maro, Wilbrod Saganda, Elizabeth A. Reddy

**Affiliations:** 1 Duke Global Health Institute, Duke University, Durham, North Carolina, United States of America; 2 Division of Infectious Diseases and International Health, Department of Medicine, Duke University Medical Center, Durham, North Carolina, United States of America; 3 Department of Pathology, Duke University Medical Center, Durham, North Carolina, United States of America; 4 Centre for International Health, Dunedin School of Medicine, University of Otago, Dunedin, New Zealand; 5 Kilimanjaro Christian Medical Centre, Tumaini University, Moshi, Tanzania; 6 Kilimanjaro Christian Medical College, Tumaini University, Moshi, Tanzania; 7 Mawenzi Regional Hospital, Moshi, Tanzania; KEMRI- Wellcome Trust Research Programme, Kenya

## Abstract

**Objective:**

In 2010, the World Health Organization (WHO) published updated guidelines emphasizing and expanding recommendations for a parasitological confirmation of malaria before treating with antimalarials. This study aimed to assess differences in historic (2007–2008) (cohort 1) and recent (2011–2012) (cohort 2) hospital cohorts in the diagnosis and treatment of febrile illness in a low malaria prevalence area of northern Tanzania.

**Materials and Methods:**

We analyzed data from two prospective cohort studies that enrolled febrile adolescents and adults aged ≥13 years. All patients received quality-controlled aerobic blood cultures and malaria smears. We compared patients' discharge diagnoses, treatments, and outcomes to assess changes in the treatment of malaria and bacterial infections.

**Results:**

In total, 595 febrile inpatients were enrolled from two referral hospitals in Moshi, Tanzania. Laboratory-confirmed malaria was detected in 13 (3.2%) of 402 patients in cohort 1 and 1 (0.5%) of 193 patients in cohort 2 (p = 0.041). Antimalarials were prescribed to 201 (51.7%) of 389 smear-negative patients in cohort 1 and 97 (50.5%) of 192 smear-negative patients in cohort 2 (p = 0.794). Bacteremia was diagnosed from standard blood culture in 58 (14.5%) of 401 patients in cohort 1 compared to 18 (9.5%) of 190 patients in cohort 2 (p = 0.091). In cohort 1, 40 (69.0%) of 58 patients with a positive blood culture received antibacterials compared to 16 (88.9%) of 18 patients in cohort 2 (p = 0.094). In cohort 1, 43 (10.8%) of the 399 patients with known outcomes died during hospitalization compared with 12 (6.2%) deaths among 193 patients in cohort 2 (p = 0.073).

**Discussion:**

In a setting of low malaria transmission, a high proportion of smear-negative patients were diagnosed with malaria and treated with antimalarials despite updated WHO guidelines on malaria treatment. Improved laboratory diagnostics for non-malaria febrile illness might help to curb this practice.

## Introduction

Fever is a common symptom among adults seeking healthcare in sub-Saharan Africa [1] and has a broad differential diagnosis [2]. Febrile illness is frequently misdiagnosed as malaria, particularly in areas of low malaria endemicity [3], [4], [5]. Inpatients with fever are often empirically treated with antimalarials, leaving bacterial infections undiagnosed and untreated [3], [6], [7]. In patients with non-malarial causes of severe febrile illness, failure to treat these alternative causes is associated with poor patient outcomes [3].

In 2010, the World Health Organization (WHO) updated its guidelines on malaria diagnosis, increasing the strength of the recommendation for parasitologic diagnosis of malaria across all age groups and in all levels of malaria transmission intensity. The reported aims of these changes included “prevention of unnecessary use of antimalarials” and “identification of parasite-negative patients in whom another diagnosis must be sought” [8].

There are many possible reasons for misattribution of bacterial febrile illness as malaria [9]. In sub-Saharan Africa, clinical laboratories often lack the facilities to provide quality malaria smears or blood cultures [10], [11], [12], contributing to malaria overdiagnosis [9]. In addition, a focus on malaria during clinical training and pressure to conform to expectations of work colleagues contribute to the overemphasis on malaria diagnosis and treatment [9]. Past studies have shown that management of malaria can improve with focused training, but that the effects diminish with time [13].

We studied the diagnosis and treatment of febrile illness in an area with low malaria endemicity by retrospectively examining diagnostic results and treatment decisions for two cohorts of adult inpatients [14] admitted to hospital between 2007–2008 (cohort 1), and 2011–2012 (cohort 2). The updated WHO recommendations on malaria were released after the completion of cohort 1 and before the beginning of cohort 2 [8], and the data from cohort 1 (which demonstrated invasive bacterial infections and bacterial zoonoses to be several-fold more prevalent than malaria) [15], [29] were disseminated to care providers at the participating hospitals at annual clinical conferences. This study aimed to examine the degree to which management of adult febrile inpatients changed before and after the publication of the updated WHO guidelines [8]. Specifically, we assessed whether treatment of malaria in smear-negative patients persisted in a low malaria-prevalence setting [14] and whether there were changes in the diagnosis and treatment of presumed or known bacterial infections.

## Materials and Methods

### Setting

Moshi, Tanzania is a town of 144,000 inhabitants and the administrative center of the northern region of Kilimanjaro (pop. 1.37 million) [15]. This area of northern Tanzania has a long rainy period between March and May and a short rainy period between October and December [16]. Moshi is located at about 890 m above mean sea level and malaria transmission is low [14]. According to data from 2007, the HIV prevalence in the Kilimanjaro region is 1.9% [17].

Kilimanjaro Christian Medical Centre (KCMC) is the referral hospital for Tanzania's northern zone and has 457 inpatient beds. KCMC serves a catchment area that includes the regions of Kilimanjaro, Arusha, Tanga, Manyara, and Singida. Mawenzi Regional Hospital (MRH) has 360 inpatient beds and serves as the regional hospital for Kilimanjaro.

Routine diagnostic tests to determine the cause of febrile illness were limited at both hospitals. Automated complete blood counts (CBC) with differential were available at both hospitals, as were gram stains of cerebrospinal or abscess fluid. Outside of the research study, both hospitals provided malaria smears; neither hospital offered malaria rapid diagnostic tests. No other rapid tests (i.e. dengue, cryptococcal antigen) were available at either hospital. Blood cultures were not available outside of the research study. *Brucella* serology was available at KCMC but not MRH, and computerized tomography (CT) scanning was available on a limited basis at KCMC but was out of the financial reach of many patients.

### Participants

We analyzed data from two prospective cohort studies designed to elucidate the causes of fever among hospitalized patients in Moshi. Cohort 1 enrolled adolescents and adults aged ≥13 years from 17 September 2007 to 31 August 2008. Cohort 2 enrolled the same age group starting on 26 September 2011 at MRH and on 2 February 2012 at KCMC. Enrollment was ongoing during the preparation of this manuscript. We excluded cohort 2 patients enrolled before 3 October 2011 or after 27 September 2012 to maintain cohort lengths of similar duration. As a result, enrollment at KCMC occurred for approximately seven months (2 February 2012 to 27 September 2012) in cohort 2.

In cohort 1, patients with oral temperatures ≥38.0°C were eligible to participate. In cohort 2, patients with subjective fevers or temperatures ≥38.0° were invited to participate. In cohort 2, temperature was either tympanic (88.2%) or axillary (11.8%). For this analysis, we only included patients with measured temperatures ≥38.0°.

Trained clinical officers obtained a clinical history and physical examination on consenting patients using a standardized clinical review form. They also collected information on treatment received prior to admission and past HIV testing results. Health care providers from the two hospitals assigned patients a preliminary diagnosis before test results were available. This analysis does not take into account the results of specific tests performed by treating clinicians outside of the research study, including malaria smears that may have been performed as part of routine care at the participating hospitals' clinical laboratories. All malaria results reported herein were conducted in the study research laboratory (described below).

After skin cleansing with isopropyl alcohol and povidone iodine, blood was drawn for aerobic blood culture and malaria smear. In the remainder of this manuscript, we refer to positive and negative peripheral malaria smears as smear-positive and smear-negative. Additional laboratory evaluations were performed in cohort 1 [18], [19], [20], [21], [22], but these were excluded from the analyses for purposes of comparability since they were not performed in cohort 2. Health care providers were provided with the immediate results of all laboratory investigations to inform clinical decisions. In both cohorts, discharge forms were completed to include information on patient outcomes, in-hospital management, and discharge diagnoses.

### Laboratory Methods

All research samples were processed in the Kilimanjaro Clinical Research Institute (KCRI) Biotechnology Laboratory. The laboratory is known in the region for its high quality results and at the time of publication it is the only local laboratory which participates fully in international external quality assurance programs. These programs include College of American Pathologists and One World Accuracy, which include regular parasite surveys in order to meet standards for diagnosis of malaria films. All laboratory investigations were conducted according to Good Clinical Laboratory Practices standards.

Thick and thin blood films were stained with Giemsa and examined for blood parasites using oil immersion microscopy. Standard methods were used to determine parasite density [23] and smear-confirmed malaria was defined as the presence of any asexual parasites per 200 leucocytes. Blood culture bottles were weighed before and after inoculation. An adequate culture volume was defined as recommended volume ±20% (8 mL–12 mL). Standard aerobic culture bottles were loaded into the BacT/ALERT 3D Microbial Detection system (bioMérieux, Durham, NC, USA) and incubated for 5 days. Standard methods were used to identify bloodstream isolates [24]. We defined contaminants as bacterial species usually associated with the skin flora and rarely implicated as causes of true bacteremia. Contaminant species included but were not limited to coagulase negative *Staphylococci*, *Streptococcus viridans*, and *Bacillus* spp [25]. Cohort 1 received HIV-1 testing as described in a previous study [18].

### Presentation of Cohort 1 Results

The results of cohort 1 analyses were published and heads of department (medicine and pediatrics at KCMC and chief of staff at MRH) were authors [18], [19], [20], [21], [22]. Data on invasive infections were presented several times for two years at grand rounds at both hospitals and discussed regularly at research forums. These presentations highlighted the low incidence of malaria smear positivity among febrile inpatients and the comparably high incidence of bacterial bloodstream infections. No new, locally-introduced patient management guidelines for febrile illness were in place or introduced as a result of cohort 1 results.

### Statistical Analysis

Data from the standardized clinical review forms were entered into an Access database (Microsoft, Redmond, WA, USA) through the Cardiff Teleform system (Cardiff, Highland Park, IL, USA). We carried out a Kruskal-Wallis test to assess differences in continuous responses. Pearson's chi-squared test was used to compare categorical and binary responses. A multivariate logistic regression model, adjusted for applicable covariates, was used to look for associations with patient mortality. Statistical analyses were carried out using STATA version 12 (Stata Corp LP, College Station, TX, USA). All statistical tests were 2-sided and used probability values (p-values) of 0.05.

To determine which participants might require antibacterial treatment based on available data, three physicians (HB, MR, ER) were independently provided a list of all the discharge diagnoses from study participants. Those diagnoses deemed to require antibacterials by all three physicians were considered to have an expert recommendation for antibacterials. Based on this assessment, we deemed the following diagnoses as having an expert recommendation for antibacterials: abscess, adenitis, brucellosis, cellulitis, endocarditis, enteric fever, meningitis (excluding cryptococcal meningitis), pneumonia, septicemia, tuberculosis, and urinary tract infection. In addition, patients reporting symptoms which would prompt treatment with antibacterials according to the Integrated Management of Adult and Adolescent Illness (IMAI) were also considered to require treatment with antibacterials; these included patients with history of convulsions or stiff neck in the setting of fever [26]. Finally, patients with documented bacteremia were considered to require antibacterials.

Because HIV testing was not performed for every patient in cohort 2, we carried out a sensitivity analysis adjusting for various levels of HIV prevalence and its effect on bacteremia prevalence. This analysis estimated the degree to which changes in baseline HIV infection might have contributed to differences in the prevalence of bacteremia. The adjusted proportion of patients with bacteremia was calculated assuming (1) that the HIV prevalence in cohort 1 was the same as the HIV prevalence at admission (i.e. that the only HIV-seropositive patients were those who tested positive prior to admission) and (2) that the prevalence was equal to the prevalence in cohort 1 (39.0%).

### Research Ethics

The two studies were independently approved by the KCMC Research Ethics Committee, the Tanzania National Institutes for Medical Research National Research Ethics Coordinating Committee, and the Duke University Medical Center Institutional Review Board. All participants, or their parent or guardian in the case of participants <18 years of age, provided written informed consent.

## Results

### Baseline Characteristics of Cohorts

**Figure 1:**
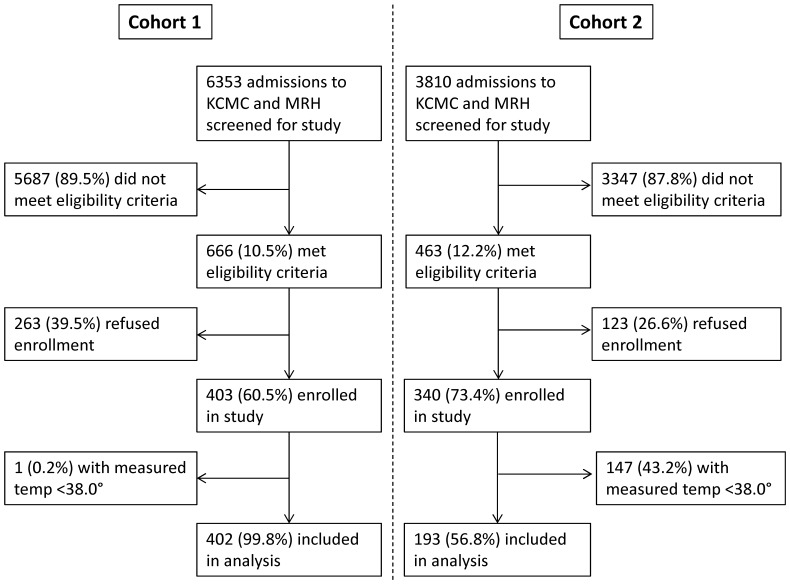
Patient Flow for febrile adults in cohort 1 (2207–2008) and cohort 2 (2011–2012) admitted to Mawenzi Regional Hospital (MRH) and Kilimanjaro Christian Medical Center (KCMC).

A total of 6353 patients were screened for enrollment in cohort 1 and 403 (6.3%) were enrolled; 3810 were screened for entry into cohort 2 and 340 (8.9%) were enrolled. For this analysis, we only included the 402 patients in cohort 1 and 193 patients in cohort 2 with measured temperatures ≥38.0°. The flow of enrollment for cohort 1 and 2 is outlined in Figure 1.

Demographic and basic clinical information for the two cohorts is shown in Table 1. Patients in the two cohorts (total n = 595) had a median (range) age of 37 (13–95) years, 337 (56.6%) were female, and 258 (49.5%) came from rural villages. There were no significant differences in age, gender, or rurality between the two cohorts. In cohort 1, 231 (57.5%) were enrolled from Mawenzi Regional Hospital (MRH), a significantly lower proportion than the 138 (71.5%) enrolled from MRH in cohort 2 (p = 0.001).

**Table 1:** Baseline characteristics of febrile adults in cohort 1 (2007–2008) and cohort 2 (2011–2012) admitted to Mawenzi Regional Hospital (MRH) and Kilimanjaro Christian Medical Centre (KCMC).

	*Cohort 1*	*Cohort 2* †	*p-value* ‡
	n (%)*	n (%)*	
Age, median (range)	36.5 (13–95)	37 (13–79)	0.984
Female	217/402 (54.0)	120/193 (62.2)	0.059
MRH admissions	231/402 (57.5)	138/193 (71.5)	***0.001***
Urban	171/351 (48.7)	92/170 (54.1)	0.248
Rigors	289/399 (72.4)	115/151 (76.2)	0.377
Headache	284/398 (71.4)	111/151 (73.5)	0.616
Cough	260/400 (65.0)	111/193 (57.5)	0.078
Vomiting	140/400 (35.0)	94/193 (48.7)	***0.001***
Shortness of Breath	136/398 (34.2)	60/193 (31.1)	0.455
Fever >7 days	85/399 (21.3)	68/193 (35.2)	***<0.001***
Diarrhea	65/396 (16.4)	33/193 (17.1)	0.834
Stiff neck	29/399 (7.3)	21/151 (13.9)	***0.016***
Convulsions	24/389 (6.2)	7/193 (3.6)	***0.198***
Hemoptysis	21/398 (5.3)	5/151 (3,3)	0.333
Jaundice	7/396 (1.8)	8/151 (5.3)	***0.024***
Past HIV test	203/401 (50.6)	121/193 (62.7)	***0.006***
Past HIV-seropositive test	97/401 (24.2)	51/191 (26.7)	0.509
All confirmed HIV-seropositive	157/402 (39.1)	N/A§	N/A
Prior antimalarials	174/396 (43.9)	79/192 (41.1)	0.521
Prior antibacterials	170/398 (42.7)	93/192 (48.4)	0.190
Prior antiretroviral therapy¶	53/97 (54.6)	35/52 (67.3)	0.134
Prior SXT prophylaxis¶	52/96 (54.2)	37/51 (72.5)	***0.030***

Significant results are marked in bold.

* Denominators less than 402 (cohort 1) and 193 (cohort 2) represent missing values.

† Questions on rigors, headache, stiff neck, hemoptysis, and jaundice were added mid-way through the study period.

‡ Significance tests for comparisons between Cohort 1 and Cohort 2 determined by Kruskal-Wallis test for continuous variables and Pearson's chi-square test for categorical variables.

§ HIV testing was not routinely performed on patients in Cohort 2.

¶ Among those with previous HIV+ test.

MRH: Mawenzi Regional Hospital; HIV: human immunodeficiency virus; SXT: trimethoprim-sulfamethoxazole.

In cohort 1, 203 (50.6%) of 401 patients had a previous HIV test versus 121 (62.7%) of 193 patients in cohort 2 (p = 0.006). Previous HIV-seropositivity was reported by 97 (24.2%) of 401 patients in cohort 1 and 51 (26.7%) of 191 in cohort 2 (p = 0.509). In cohort 1, 157 (39.1%) of 402 patients tested seropositive for HIV, 60 (38.2%) of which were among patients with no previous positive result. Because provider-initiated HIV testing had been adopted in Tanzania and all hospitalized patients were recommended to obtain HIV tests [27], the study did not offer HIV testing to cohort 2 participants; however, in practice many admitted patients were not tested for HIV.

In cohort 1, 53 (54.3%) of the 97 previously HIV-seropositive patients were receiving antiretroviral therapy (ART) compared to 35 (67.3%) of 52 HIV-seropositive patients in cohort 2 (p = 0.134). However, 52 (54.2%) of 96 HIV-seropositive patients in cohort 1 were receiving trimethoprim-sulfamethoxazole (SXT) prophylactic therapy versus 37 (72.5%) of 51 patients in cohort 2 (p = 0.030).

### Diagnosis and Treatment of Malaria

Malaria smear results for the two cohorts is shown in Table 2 and information on the diagnosis and treatment of malaria is displayed in Table 3. Malaria parasites were detected on the blood smear of 13 (3.2%) of 402 patients from cohort 1 and 1 (0.5%) of 193 patients from cohort 2 (p = 0.041). In cohort 1, 110 (28.3%) of the 389 smear-negative patients received a discharge diagnosis of malaria and 44 (22.9%) of 192 smear-negative patients from cohort 2 were diagnosed with malaria (p = 0.168). After adjusting for hospital location, 27.4% of smear-negative patients in cohort 1 received a discharge diagnosis of malaria compared to 18.7% in cohort 2 (p = 0.019). Approximately half the patients in both groups were prescribed antimalarial therapy by the inpatient healthcare providers despite a negative malaria smear: 201 (51.7%) of 389 in cohort 1 versus 97 (50.5%) of 192 in cohort 2 (p = 0.794). All 14 patients with malaria parasitemia on blood smear received antimalarials.

**Table 2:** Malaria smear and blood culture results of febrile adults in cohort 1 (2007–2008) and cohort 2 (2011–2012) admitted to Mawenzi Regional Hospital (MRH) and Kilimanjaro Christian Medical Centre (KCMC).

	*Cohort 1*	*Cohort 2*	*p-value* †
	n (%)*	n (%)*	
Malaria smear positive	13/402 (3.2)	1/193 (0.5)	***0.041***
Adequate blood volume for culture	365/401 (91.0)	152/190 (80.0)	***<0.001***
Bacterial culture positive	58/401 (14.5)	18/190 (9.5)	0.091
Bacterial culture positive (adjusted %)‡	13.8	9.8	0.194
Positive cultures arriving in time to influence clinical decisions§	43/58 (74.1)	17/18 (94.4)	0.065

Significant results are marked in bold.

* Denominators less than 403 (cohort 1) and 340 (cohort 2) represent missing values (except for culture arrival in time to influence clinical decisions).

† Significance tests for comparisons between Cohort 1 and Cohort 2 determined by 2-sample t-test for continuous variables and Pearson's chi-square test for categorical variables.

‡ Adjusted for adequate blood volume for culture, previous HIV testing, prior SXT prophylaxis, hospital location, and rurality.

§ Culture results received at least one day before patient discharge or death.

**Table 3:** Diagnoses, treatments, and outcomes of febrile adults in cohort 1 (2007–2008) and cohort 2 (2011–2012) admitted to Mawenzi Regional Hospital (MRH) and Kilimanjaro Christian Medical Centre (KCMC).

	*Cohort 1 (unadjusted)*	*Cohort 2 (unadjusted)*	*p-value* *	*Cohort 1 (adjusted)*	*Cohort 2 (adjusted)*	*p-value* *
	n (%)	n (%)		%	%	
Days in hospital median (range)	5 (1–300)	5 (1–44)	***0.134***	N/A	N/A	N/A
Malaria preliminary diagnosis†	150/402 (37.3)	61/193 (31.6)	0.173	37.3	27.6	***0.020***
Malaria discharge diagnosis†	122/402 (30.3)	45/193 (23.3)	0.074	29.3	18.8	***0.005***
Malaria smear-negative diagnosed with malaria†	110/389 (28.3)	44/192 (22.9)	0.168	27.4	18.7	***0.019***
Malaria smear-negative treated with antimalarials†	201/389 (51.7)	97/192 (50.5)	0.794	53.5	46.4	0.132
Malaria smear-negative diagnosed with malaria given antibacterials†	50/110 (45.5)	29/44 (65.9)	***0.022***	45.2	66.5	***0.017***
Malaria smear-negative treated with antimalarials given antibacterials†	135/201 (67.2)	79/97 (81.4)	***0.010***	66.9	82.6	***0.003***
Preliminary diagnosis with expert recommendation for antibacterials†	131/402 (32.6)	70/193 (36.3)	0.374	32.6	36.2	0.389
Discharge diagnosis with expert recommendation for antibacterials†	108/402 (26.9)	76/193 (39.4)	***0.002***	27.0	38.8	***0.004***
Patients with indication for antibacterials‡	164/402 (40.8)	98/193 (50.8)	***0.022***	39.1	48.4	***0.032***
Indication for antibacterials treated with antibacterials†	135/164 (82.3)	94/98 (95.9)	***0.001***	82.7	96.4	***<0.001***
Bacteremic treated with antibacterials†	40/58 (69.0)	16/18 (88.9)	0.094	68.9	89.5	0.061
Antibacterial prescription for bacteremic patients with culture results arriving in time to influence clinical decisions	31/40 (77.5)	15/17 (88.2)	0.347	§	§	§
Mortality¶	43/399 (10.8)	12/193 (6.2)	0.073	7.4	5.6	0.371

Significant results are marked in bold.

* Significance tests for comparisons between cohorts determined by Kruskal-Wallis test for continuous variables and Pearson's chi-square test for categorical variables.

† Adjusted for hospital location.

‡ Presenting symptoms of stiff neck or convulsions, positive cultures arriving in time to influence clinical decisions or discharge diagnosis with strong indication for antibacterials.

§ Unable to calculate adjusted means because of small sample size.

¶ Adjusted for hospital location and known HIV-serostatus.

Among smear-negative patients treated with antimalarials, antibacterials were prescribed to 135 (67.2%) of 201 patients in cohort 1 and 79 (81.4%) of 97 patients in cohort 2 (p = 0.010). Considering the two cohorts combined, antibacterials were prescribed to 214 (71.8%) of the 298 smear-negative patients treated with antimalarials compared to 231 (88.5%) of 261 smear-negative patients who did not receive antimalarials (p<0.001).

### Diagnosis and Treatment of Bacterial Infections

Blood culture results for the two cohorts are shown in Table 2. Among cohort 1 patients, 365 (91.0%) of 401 had an adequate blood volume for culture compared to 152 (80.0%) of 190 patients in cohort 2 (p<0.001). Bacteremia with a recognized pathogen was detected in 58 (14.5%) of 401 patients in cohort 1 and 18 (9.5%) of 190 patients in cohort 2 (p = 0.091); this difference was similar after adjusted analysis assuming the same HIV prevalence in the two cohorts. In the analysis controlling for differences in numbers of enrolled patients from KCMC versus MRH, the adjusted percentage of bacteremic patients was 13.9% in cohort 1 compared to 7.9% in cohort 2 (p = 0.025).

Information on the diagnosis and treatment of bacterial infections is shown in Table 3. In cohort 1, 40 (69.0%) of 58 bacteremic patients received antibacterials compared to 16 (88.9%) of 18 bacteremic patients in cohort 2 (p = 0.094). After adjusting for hospital location, 68.9% of bacteremic patients in cohort 1 received antibacterials compared to 89.5% in cohort 2 (p = 0.061). The results from 43 (74.1%) of the 58 positive cultures in cohort 1 and 17 (94.4%) of the 18 positive cultures in cohort 2 were available at least 24 hours prior to patient discharge or death, which was our definition of arrival in time to influence clinical decisions (p = 0.065). When limiting the analysis to cultures arriving in time to influence clinical decisions, 31 (77.5%) of 40 bacteremic patients received antibacterials in cohort 1 versus 15 (88.2%) of 17 in cohort 2 (p = 0.347).

In cohort 1, 164 (40.8%) of 402 patients and, in cohort 2, 98 (50.8%) of 193 patients had an indication for antibacterials (i.e. symptoms of stiff neck or convulsions, bacteremia arriving at least 24 hours before discharge or death, and discharge diagnosis with an expert recommendation for antibacterials) (p = 0.022). Among patients with a strong indication for antibacterials based on presenting symptoms, culture results, or discharge diagnoses, antibacterials were provided to 135 (82.3%) of 164 in cohort 1 and 94 (95.9%) of 98 in cohort 2 (p = 0.001).

### Patient Mortality

Information on patient mortality in the two cohorts is shown in Table 3. In cohort 1, 43 (10.8%) of the 399 patients with known outcomes died during hospitalization compared to 12 (6.2%) deaths among 193 patients in cohort 2 (p = 0.073). Mortality was associated with known HIV-seropositivity (r = 0.083, p = 0.003) and admission to KCMC (r = −0.097, p<0.001). The difference in mortality between cohorts remained non-significant after adjusting for these variables (p = 0.149). All of the patients who died were malaria smear-negative.

## Discussion

Our results indicate that in an area of low malaria prevalence, febrile illness was often diagnosed and treated as malaria. Such management did not change from cohort 1 to cohort 2 despite increasingly greater emphasis from the WHO to limit antimalarial treatment to laboratory-confirmed malaria cases, low malaria prevalence throughout the study periods, and dissemination of information gleaned from cohort 1 to healthcare providers. Given our conservative definition of laboratory-confirmed malaria [28] and the rarity of smear-negative malaria, it is unlikely that cases of clinical malaria were missed. Approximately one quarter of patients with negative blood smears received a discharge diagnosis of malaria and more than half of smear-negative patients received antimalarial treatment. While the treatment of malaria in smear-negative patients remained stable, the analysis adjusted for hospital location and objective fever suggests that the clinical diagnosis of malaria in smear-negative patients decreased in cohort 2.

One-tenth of febrile inpatients had positive blood cultures and bacteremia was seven-times more common than laboratory-confirmed malaria. Nevertheless, malaria was diagnosed as often as bacterial infections with an expert recommendation for antimicrobial treatment. In addition, while every smear-positive patient was treated with antimalarials, nearly 15% of patients with expert recommendations for antibacterial treatment did not receive antibacterials. However, more patients with a strong indication for antibacterials received them in cohort 2 compared with cohort 1. Among all patients, smear-negative patients treated with antimalarials were less likely to receive antibacterials. These findings correspond with earlier reports showing an association between use of antimalarials in smear-negative patients and non-treatment with antibacterials [3], and highlight one of the most important reasons to address and alter the practice.

Our study demonstrated a non-significant trend towards a decline in prevalence of bacteremia and mortality between the two cohorts. If HIV prevalence was indeed lower in cohort 2, this may have influenced such findings. In addition, an increased proportion of patients in cohort 2 reported use of antiretroviral medications and TMP/SMX prophylaxis. Alterations of other environmental or community risk factors or normal fluctuations in disease patterns may have contributed to this trend.

While enhancing local standards of care to include availability of blood culture and antimicrobial susceptibility would greatly improve the accurate diagnosis of local pathogens, other infectious diseases such as leptospirosis, rickettsioses, and arboviral infections were found to be common causes of febrile illness in cohort 1 [29]. The overdiagnosis of such illnesses as clinical malaria may be difficult to curb until increased awareness and, optimally, affordable and accurate diagnostics for such illnesses are available.

This study had the strength of examining two relatively large cohorts of patients with febrile illness from the same two hospitals before and after changes in the WHO guidelines for malaria diagnosis and treatment [8]. Differences in the HIV testing between the two cohorts were relative weaknesses which we attempted to address with adjusted analyses. We also acknowledge that any false-positive malaria smear results that may have been provided locally were not accounted for in this analysis and such results could have driven clinicians to over-treat for malaria. In addition, it is likely that our definition for having an indication for antibacterials did not capture patients in the IMAI severe illness category [26]. Therefore, it is likely that we underestimated the proportion of patients with an indication for antibacterial treatment.

Taken together, these results indicate that updates in WHO guidelines and a consistent emphasis on parasitologic diagnosis have not been sufficient to change the practice of frequently diagnosing and treating “clinical” malaria among hospitalized adults in northern Tanzania. Laboratory results that are not available in time to affect clinical decisions, lack of confidence in results, pressure to conform to previous standards of care, inadequate dissemination of updated WHO guidelines for malaria treatment, and a lack of knowledge of other possible causes of febrile illness may all contribute to ongoing malaria overdiagnosis [9]. Health care providers might not be recognizing signs of severe illness in febrile patients [26]. Novel or more directed efforts to change prescribing patterns may be needed to alter the status quo [13]. Estimating the direct costs of malaria overtreatment could provide further motivation to re-address this practice. Finally, while this study has highlighted the importance of considering and treating bacterial infections in hospitalized febrile patients in our setting, risks of antimicrobial resistance with empiric use of antibacterials are not to be ignored. Improved laboratory diagnostics for non-malaria febrile illness in resource limited settings is likely to be key in encouraging appropriate treatment.
